# Transcript Analysis Reveals a Specific HOX Signature Associated with Positional Identity of Human Endothelial Cells

**DOI:** 10.1371/journal.pone.0091334

**Published:** 2014-03-20

**Authors:** Mark Toshner, Benjamin J. Dunmore, Eoin F. McKinney, Mark Southwood, Paola Caruso, Paul D. Upton, John P. Waters, Mark L. Ormiston, Jeremy N. Skepper, Gerard Nash, Amer A. Rana, Nicholas W. Morrell

**Affiliations:** 1 Department of Medicine, University of Cambridge, Addenbrooke's Hospital, Cambridge, United Kingdom; 2 Papworth Hospital, Cambridge, United Kingdom; 3 Department of Physiology and Neuroscience, University of Cambridge, Cambridge, United Kingdom; 4 School of Clinical and Experimental Medicine, Birmingham University, Birmingham, United Kingdom; University of Miami School of Medicine, United States of America

## Abstract

The endothelial cell has a remarkable ability for sub-specialisation, adapted to the needs of a variety of vascular beds. The role of developmental programming versus the tissue contextual environment for this specialization is not well understood. Here we describe a hierarchy of expression of HOX genes associated with endothelial cell origin and location. In initial microarray studies, differential gene expression was examined in two endothelial cell lines: blood derived outgrowth endothelial cells (BOECs) and pulmonary artery endothelial cells. This suggested shared and differential patterns of HOX gene expression between the two endothelial lines. For example, this included a cluster on chromosome 2 of HOXD1, HOXD3, HOXD4, HOXD8 and HOXD9 that was expressed at a higher level in BOECs. Quantative PCR confirmed the higher expression of these HOXs in BOECs, a pattern that was shared by a variety of microvascular endothelial cell lines. Subsequently, we analysed publically available microarrays from a variety of adult cell and tissue types using the whole “HOX transcriptome” of all 39 HOX genes. Using hierarchical clustering analysis the HOX transcriptome was able to discriminate endothelial cells from 61 diverse human cell lines of various origins. In a separate publically available microarray dataset of 53 human endothelial cell lines, the HOX transcriptome additionally organized endothelial cells related to their organ or tissue of origin. Human tissue staining for HOXD8 and HOXD9 confirmed endothelial expression and also supported increased microvascular expression of these HOXs. Together these observations suggest a significant involvement of HOX genes in endothelial cell positional identity.

## Introduction

HOX genes are homeobox-containing genes conserved across mammalian species, which encode transcription factors involved in the determination of positional identity. Although the role of HOX genes in this process is well described, they also play important roles in adult tissues. In studies in adult fibroblasts, and more recently mesenchymal cells, from different locations in adult tissues, patterns of HOX expression are retained *in vitro* when cells are removed from their contextual environmental cues [1], [2], [3]. Dysregulated HOX expression in cancers also suggests a retained cellular identity switch driving loss of normal phenotype [4], [5], [6].

During development the vascular bed undergoes a highly patterned programme of vasculogenesis and angiogenesis. The adult endothelial cell phenotype varies according to the local requirements placed on it by individual organs. This is of particular importance for regenerative medicine, where attempts to develop viable organs or bioprostheses rely on the production of an appropriate and functional vascular bed. Several cells types have been suggested as progenitors for endothelial cell therapeutics and engineering, perhaps most promisingly the blood-derived outgrowth endothelial cell (BOEC) [7]. The BOEC can be autologously generated from a peripheral blood sample and has the capacity for rapid expansion. It is thought to be of a “progenitor” phenotype and therefore has potential in organ vascularisation and therapeutic indications. Here we report a hierarchy of HOX gene signatures specific to endothelial cells, which we first identified in BOECs. Further comparison of transcriptomes of endothelial cells from diverse vascular beds confirmed that the HOX gene signature predicts the positional identity of endothelial cells, providing insights into the potential role of these genes in endothelial differentiation.

## Methods

### Isolation and Culture of BOECs

PBMNCs were isolated from 50 mls of blood by Ficoll density gradient centrifugation and plated onto flasks coated in rat-tail collagen (BD Biosciences, Bedford, MA) in endothelial selective media (EGM2, Lonza Biologics, Slough, UK) as previously described [8]. Full informed written consent was obtained and the Huntington Local Research Ethics Committee approved these studies. The only change to the original protocol was the initial generation of cells included the use of 20% ES screened media (Hyclone, UK).

### Human tissue studies

Embryonic tissue was sourced from the MRC/Wellcome Trust-funded Human Development Biology Resource, Newcastle upon Tyne, UK, with full informed written consent and ethical approval from the Newcastle Upon Tyne Joint Research Ethics Committee. Tissue from later gestational ages was obtained from the MRC Tissue Bank, Division of Investigative Science, Hammersmith Hospital and the Institute of Child Health, London, UK, and used with the approval of the local ethics committee. Tissue consisted of serialized 4 μm sections of formalin-fixed, paraffin-embedded whole human embryo mounts and fetal lung, classified by approximate gestational age in days. Tissues were categorized as follows: embryonic (<day 49); pseudoglandular (days 49–112); canalicular (days 112–156); saccular (days 156–266/term); and developed lung.

### Immunostaining and confocal microscopy

Tissue blocks were sectioned (4 mm) with a microtome (Leica Micro- systems, Milton Keynes, UK), placed onto poly-L-lysine–coated slides, dried at 60°C for 1 hour, dewaxed and dehydrated through graded alcohols. Slides were microwaved for 30 minutes and incubated in proteinase K (Dako, Ely, UK) for 10 minutes. Endogenous tissue peroxidase was bound with hydrogen peroxidase blocking solution (Dako). All primary antibodies were incubated for 1 hour at RT. The primary antibodies were polyclonal rabbit anti-human HOXD8, HOXD9 (Abcam, Cambridge, UK). For control purposes in antibodies for which peptide was not available the primary was omitted. Primary antibodies were labeled using goat anti rabbit dextran coupled peroxidase and visualized with 3,3- diaminobenzidine hydrochloride. Sections were counterstained in Carazzi's hematoxylin, mounted in DPX (VWR/Merck, Lutterworth, UK) and examined by light microscopy. For fluorescence immunostaining, cells were permeabilized in absolute methanol at -200C for 5 minutes, washed, and incubated at 4–80C overnight in primary antibody (rabbit anti-human fibronectin, vWF, CD31 and CD146 (Abcam)) and subsequently in secondary antibody; goat anti-rabbit Texas red, horse anti-mouse fluorescein isothiocyanate (FITC) (Vector Laboratories Ltd, Peterborough, UK), and then in TO-PRO-3 iodide (Molecular Probes, Eugene, OR) for nuclear staining, before mounting with VECTASHIELD. Isotype controls were employed to verify specificity of staining. Slides were viewed with a Leica confocal laser-scanning microscope.

### Electron microscopy

BOECs were grown in media as above and fixed in 4% glutaraldehyde containing 2 mmol/L CaCl2 in 0.1 mol/L PIPES buffer (pH 7.4), supplemented with 0,33% H2O2 immediately before use. Samples were incubated for 4 hours at 4°C, washed twice in 0.1 mol/L PIPES (pH 7.4) and kept at 4°C. After post-fixation in 1% osmium ferricyanide for 1 hour, samples were rinsed 3 times in DIW and incubated in 2% uranyl acetate for 1 hour. Then samples were rinsed in water and dehydrated in an ascending series of ethanol solutions to 100% ethanol, rinsed twice in acetonitrile and embedded in Quetol epoxy resin (9.0 g Quetol 651, 11.6 g nonenylsuccinic anhydride (NSA), 5.0 g methylnadic anhydride (MNA) and 0.5 g benzyl dimethylamine). Pellet sections were cut on a Leica Ultracut UCT and analyzed using a FEI Tecnai G^2^ operated at 120 kv. For immunogold labeling cells were fixed in 6% formaldehyde, dehydrated in ethanol and embedded in LR White acrylic resin. Primary antibodies were visualized using species specific secondary antibodies conjugated to 15 nm gold particles.

### Network formation

Matrix gel (Chemicon, CA) was seeded into the wells of a 96-well plate and incubated at 37°C for 1 hour. Cells (10,000) were suspended in 250 ml of medium supplemented with 10% FCS and seeded onto the gel plugs. All experiments were performed in quadruplicate. After 4 hours cells were visualized by phase-contrast light microscopy.

### Lumenisation Assay

BOECs were suspended in a solution of rat-tail type 1 collagen (1.5 mg/ml, BD, Franklin Lakes, NJ, USA) and human plasma fibronectin (90 ug/ml, Millipore, Billerica, MA, USA) in 25 mM HEPES and 1.5 mg/ml NaHCO_3_ buffered M199 medium at 4°C. The pH was adjusted to 7.4 using 0.1 M HCl. The suspension was pipetted into 48 well tissue culture plates and polymerised at 37°C in 350 ul per well. The gel was overlaid with 0.5 ml media.

### Flow mediated leucocyte adhesion

Primary cultures of BOECs between passages 3–6, were dissociated with trypsin/EDTA (Sigma) and seeded into rectangular glass capillaries (microslides, internal width 3 mm, depth 0.3 mm) coated with collagen/gelatin. Seeding was at a density yielding confluent monolayers within 24 h. After seeding, the microslides were placed into specially constructed glass dishes and attached to a perfusion culture system pumping a small amount of medium through the microslide for 30 s once an hour as described [9]. BOECs were cultured for 48 h and then TNF-α (100 or 1000 U/ml) was added for a further 4 h. For neutrophil preparation blood was collected from healthy volunteers into EDTA (Sarstedt Ltd., UK). Full informed written consent was obtained and ethical approval was from University of Birmingham, Life and Health Sciences Ethical Review Committee. Neutrophils were isolated as described previously [9]. Microslides containing confluent BOECs were attached to a flow system mounted on a phase contrast video microscope and neutrophils perfused at a wall shear stress of 0.1 Pa for 4 min. After bolus perfusion and a 5-min washout, the numbers of adherent neutrophils rolling, stably adherent or transmigrated were counted, and the total corrected per square millimeter per 10^6^ cells perfused.

### Microarray studies

Human pulmonary artery endothelial cells (n = 4) (Lonza) and BOECs (n = 4) were maintained in endothelial cell growth medium-2 (EGM-2) (Lonza). For the microarray study, cells were grown in serum restricted conditions in M199 (Sigma-Aldrich, UK) containing 0.1% FBS for 16 hours prior to RNA extraction. Total RNA was isolated using Trizol (Life Technologies, UK) and assessed using the NanoDrop 1000 spectrophotometer (Thermo Scientific, DE, USA). Array profiling was performed using the GeneChip Human Genome U133 Plus 2.0 Array (Affymetrix, CA, USA). Biotin-labelled aRNA was prepared using the GeneChip 3′ IVT Express kit (Affymetrix) using manufacturer's protocols. Labelled RNA was hybridised onto GeneChip microarrays as per manufacturer's protocols prior to scanning. After scanning .CEL files were imported into R and assessed using ArrayQualityMetrics in the Bioconductor package. In R using the Bioconductor package, probe level data was summarised and normalised using Robust Multi-Array Analaysis (RMA) and quantile normalisation. A t-test was used to call differentially expressed genes at each of the developmental stages (P < 0.05 after false discovery rate correction).

### Analysis of published microarrays

Publicly-available microrray datasets were imported from NCBI-GEO or ArrayExpress [10] into R using Bioconductor [11] or GenePattern [12] for further analysis.

### Comparative transcriptomics

Unfiltered normalized expression data from BOECs were imported into the GeneExpression Barcode platform and converted to binary expression calls (expressed or not) [13]. Principal components analysis of the resulting binary matrix was used to visualize proximity of the BOEC/PAEC transcriptomes to multiple other cell types included in the barcode repository [14].

### HOX-gene expression

Expression of the HOX gene family was determined by extracting data for all HOX genes present on separate platforms in each of three independent published datasets. HOX genes were not equivalently represented on all platforms. These were 1) 61 normal human cell cultures of epithelial, endothelial, and stromal cells (GSE3239, GE Codelink Human Uniset), 2) Flk^+^ and Flk^−^ murine embryonic stem cells at three timepoints during differentiation (GSE3757 [15], Agilent 011472 Mouse Development OligoArray) and 3) 53 cultured normal human vascular endothelial cell samples from 14 different tissues (GSE3268 [16], SHDD).

### Quantative real time PCR

Total RNA (including miRNAs) was extracted from cells incubated in either EGM2/10% FBS for 24 h using QIAGEN miRNeasy Mini columns with DNase digestion protocol according to the manufacturer's instructions. To assess HOX genes expression total RNA was reverse transcribed and amplified by PCR using a one-step RT-PCR kit (Access RT- PCR System; Promega, UK) with relevant upstream and downstream primers and 1.5 mM magnesium sulfate. RT-PCR reactions were amplified on a PCR Express Thermocycler (Thermo Fisher Scientific, UK) and performed on Applied Biosystems StepPlusOne RT qPC as previously described [17]. Reactions were amplified on an iCycler (Bio-Rad Laboratories, UK). The efficiency of each primer set was confirmed to be between 90 and 100% cDNA. To obtain accurate housekeeping genes cDNA samples from BOECs and endothelial cells were compared for the same gene set, and the housekeeping genes 18S rRNA, B2M and Β-actin were included on every plate as a reference when between cell type comparisons were being made and controls were normalized using the GeNorm program. When responses in individual cell types were being compared, Β-actin was used as a control reference. Specific primers used were Qiagen Quantitect for HOXD1, HOXD4, HOXD8, HOXD9, 18 s rRNA and B2M. HOXD3 (sense 5′-AATTGTGGTCACCTGGAGCCT-3′, antisense 5′-GGCCTGCTGACCCTGCTCAAA-3′) Β actin (sense 5′-GCACCACACCTTCTACAATGA-3′, antisense 5′-GTCATCTTCTCGCGGTTGGC-3′). cDNA for miRNA analysis was synthesized from total RNA using stem-loop reverse transcription primers and then assessed by RT-PCR using specific miRNA TaqMan probes according to the TaqMan miRNA Assay protocol (Applied Biosystems, Foster City, CA, USA). Results were normalized to the small nuclear RNA Rnu-48. The fold change for every miRNA expression was obtained using the 2-ΔΔ Ct method. The RT-PCRs for each miRNA were run in triplicate and results are presented as the mean ± standard error of samples.

## Results

### BOECs are phenotypically and functionally mature endothelial cells

**Figure 1:**
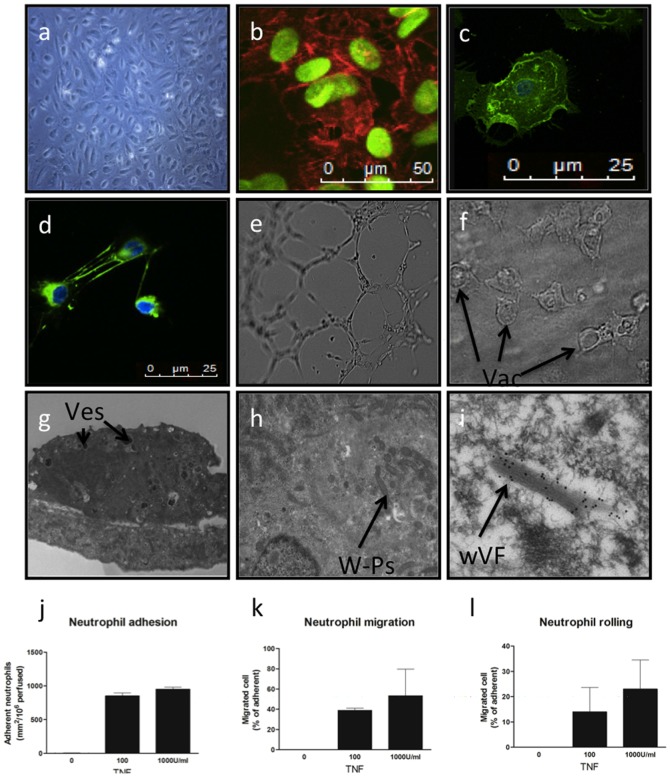
The BOEC is functionally a mature endothelial cell. a) Phase microscopy of BOECs in culture demonstrates typical endothelial “cobblestone” morphology. Confocal microscopy of BOECs with nuclear counterstaining. b) CD31 (red), c) CD146 (green), d) fibronectin (green). e) Network formation in matrix gel, f) vacoulised BOECs in fibronectin/collagen matrix. g) Electron microscopy (EM) of whole BOEC showing extensive vesicles (Ves), h) EM close up of W-P bodies, i) EM of W-P bodies with immunogold staining for vWF. j-l) Neutrophil rolling adhesion and transmigration after stimulation with TNF in a flow-based assay.

The BOEC exhibits a robust endothelial phenotype. They grow from oligoclonal colonies and form cobblestone monolayers in culture and express endothelial markers; CD31, CD146 and vWF (figure 1b,c,i). Electron microscopy confirmed that they are rich in vesicles and contain Weibel-Palade bodies, as evidenced by characteristic tubular architecture and immunogold staining for vWF (figure 1g–i). BOECs produced extracellular matrix (figure 1d) as recently described [18], and formed networks in matrigel-based assays (figure 1e). In custom-made fibronectin/collagen based 3D gels, which support viable cells for longer periods, clear evidence for vacuolization was observed, an endothelial specific process (figure 1f). BOECs support neutrophil rolling, followed by firm adhesion and transmigration when stimulated by tumour necrosis factor-α in a flow-based *in vitro* assay (figure 1j-l), a process unique to endothelial cells to our knowledge.

### Transcriptome analysis of BOECs

**Figure 2:**
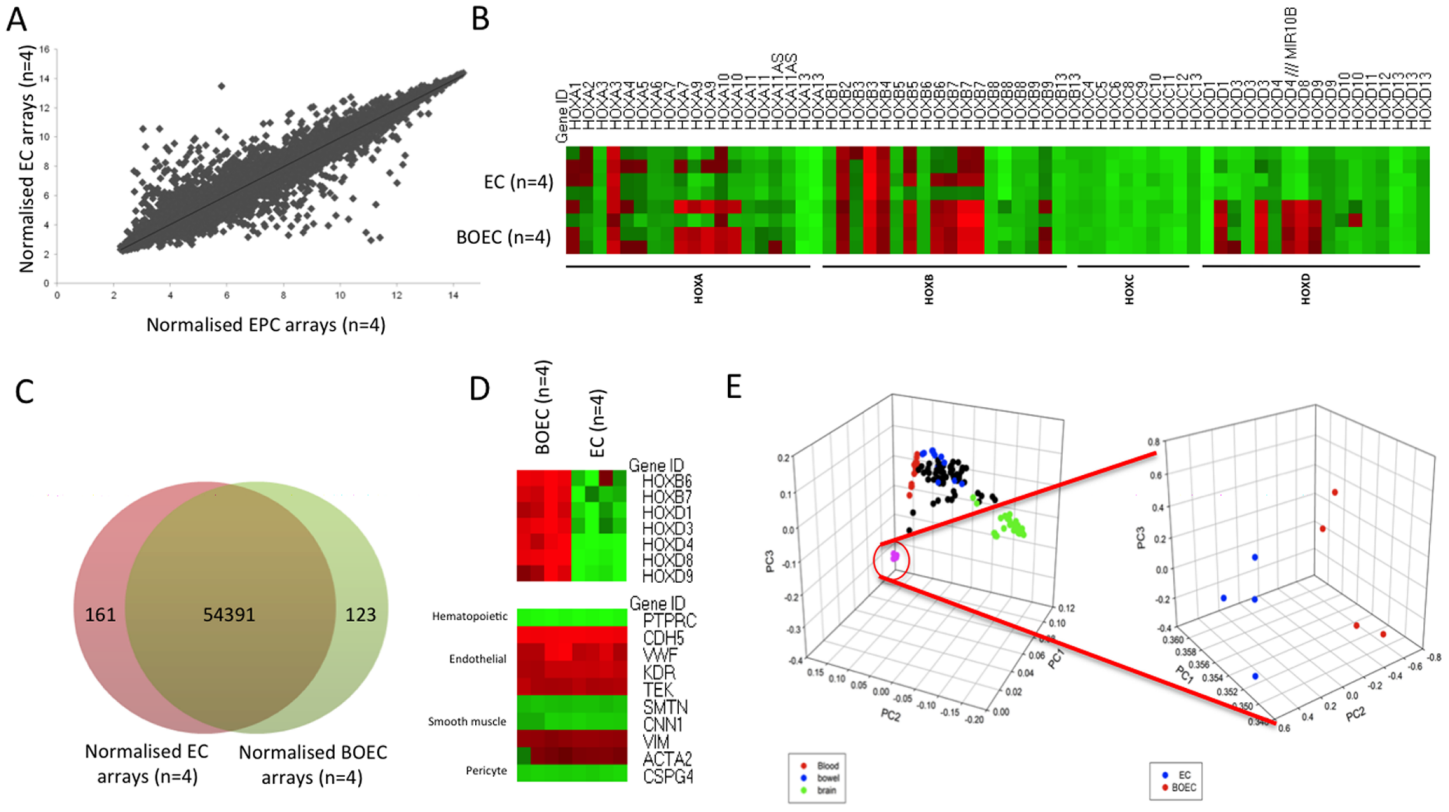
BOECs have a near identical mRNA microarray profile to PAECs but differ significantly in HOX expression. a) Microarray expression of BOECs vs PAECs (n = 4) demonstrates high concordance of gene expression b) Heat map of HOX genes in BOECs vs PAECs. c) Venn diagram showing convergence of gene expression, d) Heat maps of differentially expressed HOXs. Additional lineage associated transcripts showing endothelial expression pattern, e) PCA analysis of BOEC vs 131 tissue arrays. Principal components analysis; Graph 1-blood derived cells (red), bowel (blue), brain (green); Graph 2 PAECs (blue), BOECs (red) demonstrating similarity of BOECs and PAECs when compared to other mature cell types.

Microarray studies comparing the BOEC transcriptome with that of a differentiated adult endothelial cell line (pulmonary artery endothelial cells- PAECs) demonstrated a high degree of similarity between the 2 cell types (figure 2a,b). Using a 2 fold cutoff criteria, only 0.005% of gene transcripts were differentially expressed (figure 2c). In particular there were no differences in the expression levels of traditional endothelial genes (figure 2d). We then compared the transcriptomes of BOECs and PAECs with those from 131 other published and publicly available non-endothelial tissue and cell microarrays using principle component analysis (PCA) [13]. This approach demonstrated clustering of cells from similar lineages (blood-derived cell types highlighted in red, bowel in blue and brain in green) (figure 2e). BOECs and PAECs clustered together (figure 2e). When the BOECs and PAECs were compared in isolation there was a subtle, significant grouping of the 2 cell types apart from each other, revealing a small variance between these endothelial cell types (figure 2e). Using gene ontology analysis, the differentially expressed genes were clearly and specifically related to development and morphogenesis (table 1).

**Table 1:** Gene ontology analysis - differentially regulated genes between BOECs and PAECs using 2 fold cut-off.

	Term	Classic Fisher	ClassicKS	elimKC
1	Regionalisation	2.40E-11	0.00356	0.50407
2	Pattern specification	5.70E-11	0.00052	0.07208
3	Anterior/posterior pattern specification	8.60E-11	0.00124	0.00343
4	Embryonic skeletal development	9.20E-11	1.30E-07	0.01336
5	Embryonic skeletal morphogenesis	1.10E-10	2.30E-06	7.80E-10
6	Chordate development	4.30E-09	0.00085	0.79168
7	Skeletal system morphogenesis	4.40E-09	0.00018	0.40402
8	Embryonic development	4.90E-09	0.00077	0.6932
9	Embryonic organ morphogenesis	6.90E-08	0.00086	0.63877
10	Skeletal system development	9.00E-08	0.00019	0.06121

### The HOXD signature differentiates BOECs from large artery ECs

**Figure 3:**
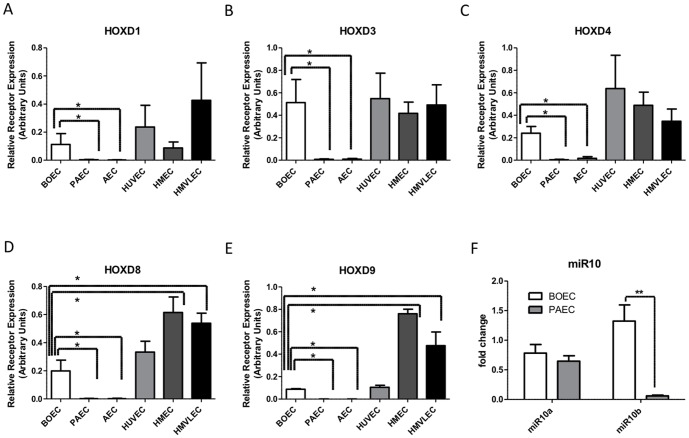
HOX expression validation by qPCR. a-e) HOXD1, D3, D4, D8 and D9 mRNA expression in BOECs, pulmonary artery ECs (PAECs), Aortic ECs (AECs), umbilical vein ECs (HUVECs), microvascular lymphatic ECs (HMECs), lung microvascular ECs (HMLECs). Expressed in relative arbitrary units. f) MicroRNA10 expression in BOECs and PAECs. Data was analyzed by ANOVA *p<0.05 **p<0.01.

Closer inspection of the genes differentially expressed between BOECs and PAECs revealed differences dominated by HOX gene networks, particularly a HOXD linearly arranged gene cluster (figure 2c,d). We confirmed the differential expression of HOXD cluster genes by qPCR (figure 3a-e). HOXD1, HOXD3, HOXD4, HOXD8 and HOXD9 were all highly expressed in BOECs compared with PAECs. HOX genes are well known as master regulators of positional identity. We therefore questioned whether the HOXD gene signature might reflect the differing origins of the endothelial cells. PAECs are derived from large conduit pulmonary arteries, whereas the origin of BOECs remains unclear. We examined transcripts from endothelial cells derived from 3 differing categories: 1) Microvascular (pulmonary-HMVLECs and lymphatic-HMECs), 2) Macrovascular (pulmonary-PAECs and aortic-AECs), 3) HUVECs. There was significantly higher expression of all HOXD genes in BOECs when compared to PAECs and AECs but no significant differences between BOECs and microvascular cells or HUVECs (figure 3a–e).

### HOX gene signatures stratify endothelial cells according to positional identity

**Figure 4:**
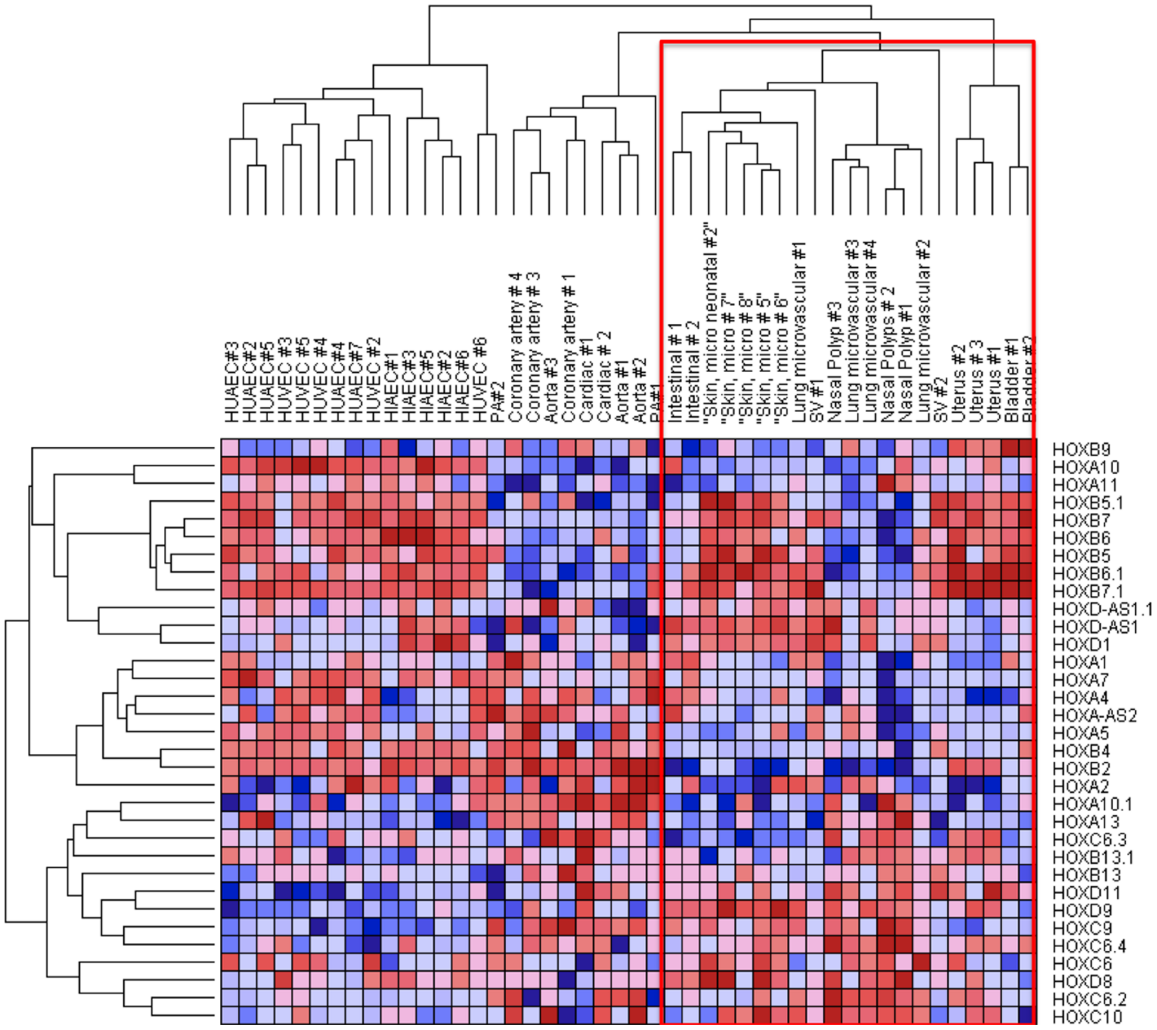
Heat map and hierarchical clustering of publically available microarray data from 53 endothelial cell types. Hierarchical analysis demonstrates that microvascular cells are ordered in hierarchies (within the red box). Adult and foetal cell lines segregate in separated hierarchies, as do cardiovascular-derived endothelial cells.

To test whether HOX genes can discriminate between macrovascular and microvascular endothelial phenotypes we interrogated publicly available and published microarrays [15], [16]. First we interrogated a dataset of endothelial cells from an expanded variety of 53 endothelial cell arrays [16]. To discriminate between our microarrays and publically available datasets our own data are presented in green/red heat maps in the figure legends, whereas published datasets are red/blue heat maps. Heat-map analysis organized endothelial cells into hierarchies notable for clustering of similar endothelial cell types. The most hierarchically distinct is the cluster of HUACs, HUVECs and HIAECs. Cardiovascular endothelial cells clustered together, as did lung microvascular and nasal, uterine and bladder. Microvascular cells are all found within one generation and segregated from the macrovascular cell types (red box) (figure 4). Moreover, endothelial cells clustered by developmental origins (figure 4). Developmentally related cell types, for example coronary, cardiac and aorta; lung and nasal; bladder and uterine; HUVEC and HUAEC, all cluster together. Thus, HOX gene expression is able to segregate macrovascular from microvascular, fetal-derived cells from adult, and clusters cells in hieriarchies corresponding to their developmental origin.

HOX gene clusters contain genes for non-coding miRNAs (microRNAs), known to be involved in feedback autoregulation of HOX genes [19]. MiR-10b locus maps within the linear gene cluster upstream of HOXD4 in the promoter region, and was highly expressed in BOECs when compared to PAECs (figure 3f). There was no difference in the expression level of the related miR-10a, which maps upstream of the HOXB4 gene in the B cluster. Of note, HOXB4 was similarly expressed in both BOECs and PAECs (figure 2c). MiR-10b has very recently been implicated in angiogenesis [20]. Therefore the HOXD linear cluster is upregulated in concert, and includes a miRNA contained within a promoter region, suggesting that the cluster may be acting as an operon under a common regulatory signal.

**Figure 5:**
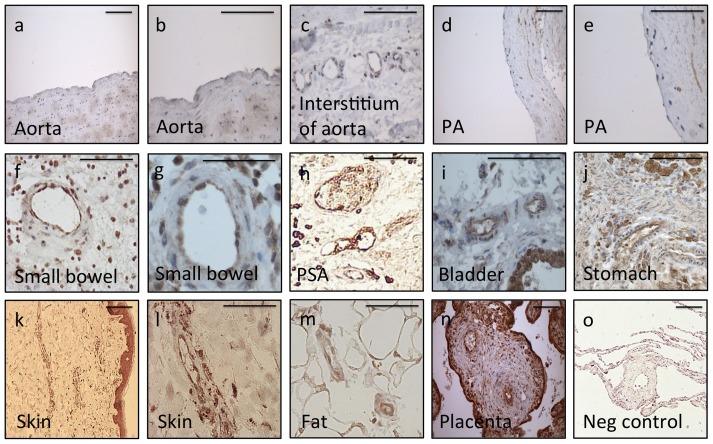
Adult human tissue immunostaining of HOXD9 confirms endothelial expression. Tissue expression of HOXD9. a) Aorta, low power (x200) and b) high power (x400), c) staining of small vessels surrounding the aorta (x630) d) Main pulmonary artery low power (x200) and e) high power (x400), f) small arteries in small bowel low power (x200) and g) high power (x630), h) small pulmonary arteries (x200), i) bladder with small arteries and epithelium visible (x630), j) stomach with small vessels and mucosal glands (x400), k) skin with microvasculature and epithelium at low power (x200) and l) skin microvasculature at high power (x400), m) small arteries within fat surrounding muscle (x400), n) placental villi with central small arteries (x200), o) negative control in pulmonary vessel (x200). Scale bars 100 μm.

To confirm the mRNA data at the level of protein expression we concentrated on HOXD8 and HOXD9, which were most highly expressed in BOECs. Immunostaining for HOXD9 shows conserved endothelial expression in small vessels from a wide variety of organ beds (figure 5). Staining was lowest in aorta (figure 5a,b) and main pulmonary artery (figure 5d,e). By comparison small vessels of the vasa vasorum of the aorta and small pulmonary vessels demonstrated more intense endothelial staining (figure 5c, h). HOXD9 was also observed in small vessels in the small bowel, stomach, bladder, skin, fat surrounding muscle and placenta (figure 5f-n). Staining within vessels was specific to the intimal layer, but was also observed in other cell types, most consistently epithelium (figure 5k,i). Similar endothelial staining was also demonstrated with HOXD8 (figure S1).

### HOX genes segregate endothelial cells from a diverse variety of cell lines and are strongly expressed during angiogenesis

**Figure 6:**
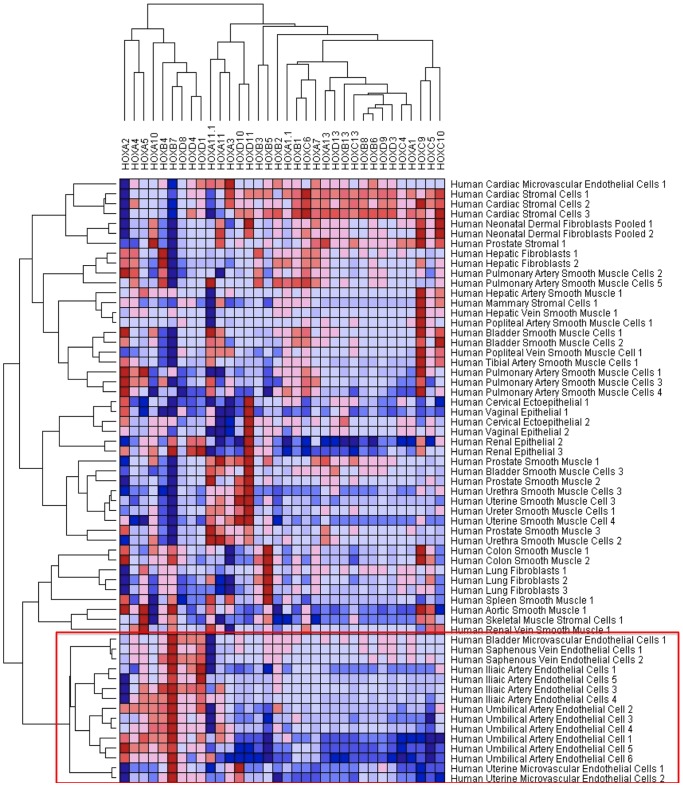
Heat map and hierarchical clustering of publically available microarray data of 61 cell types using HOX expression. Endothelial cells all cluster within the same hierarchy (within red box) with the exception of cardiac microvascular cells.

**Figure 7:**
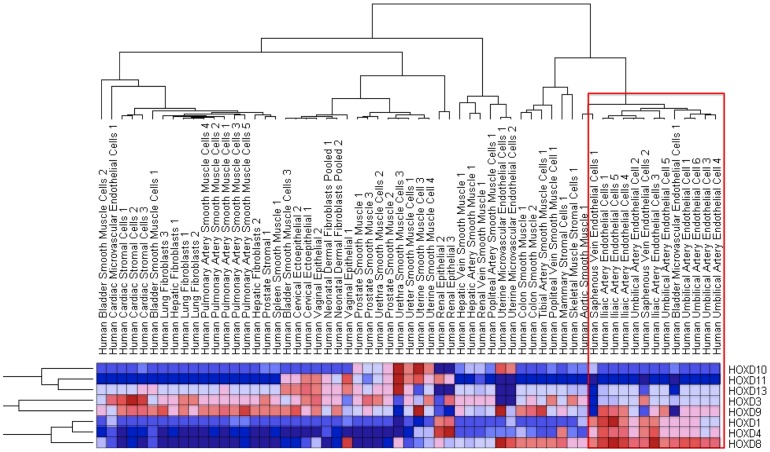
Heat map and hierarchical clustering of publically available microarray data of 61 cell types using HOXD expression only. There remains a similar endothelial hierarchy, with the exception of uterine microvascular cells.

**Figure 8:**
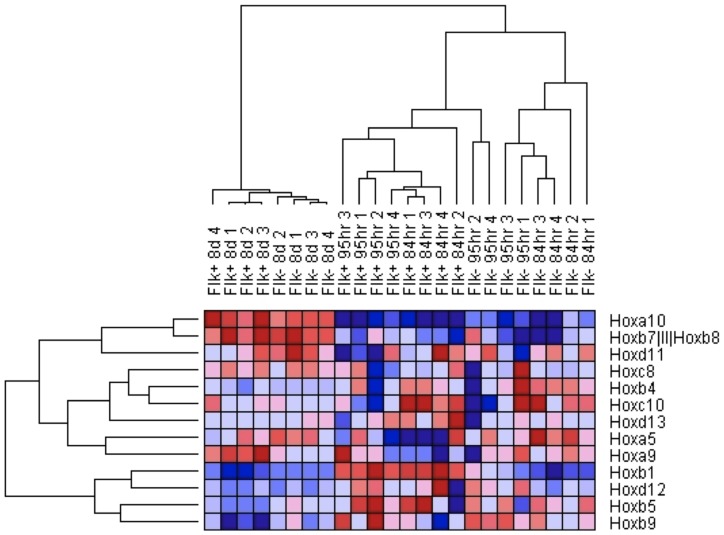
Microarray data from mouse ES cells differentiated towards endothelial phenotype in vitro also segregates according to HOX expression. Heat map and hierarchical clustering of publically available microarray data from mouse ES cells differentiated towards an endothelial lineage (Flk +ve) at 84 hrs, 95 hrs and 8 days.

To determine whether the pattern of HOX gene expression in ECs is simply related to body segmentation we examined a larger dataset including mesenchymal and ectodermally derived cells from similar segmental locations. Comparison of transcripts from 61 diverse adult human cell lines showed that endothelial cells cluster together in a distinct hierarchy (figure 6). The only exception to this was coronary artery endothelial cells, which clustered with other cardiac cells. This is consistent with current knowledge, which suggests a common epicardial origin for all cardiac cells [21]. Notably endothelial cells did not cluster with cells from similar segmental regions, suggesting a hierarchy of endothelial identities unrelated to an “organ specific” identity. Again the HOXD cluster appeared to be largely responsible for this segregating effect since a reanalysis of this dataset using only the HOXD cluster demonstrated a similar result (figure 7). To provide further insight into whether HOXD expression is developmentally programmed we analysed a dataset of mouse ES cells differentiated *in vitro* into endothelial cells [15]. Again we observed a HOX signature that discriminated between the undifferentiated cells at 4/5 days compared with the 8-day timepoint when the cells have acquired an endothelial phenotype (figure 8). Furthermore, segregating the subsets at each timepoint into FLK+ve and –ve cells, again demonstrated a clear HOX programme associated with endothelial phenotype.

**Figure 9:**
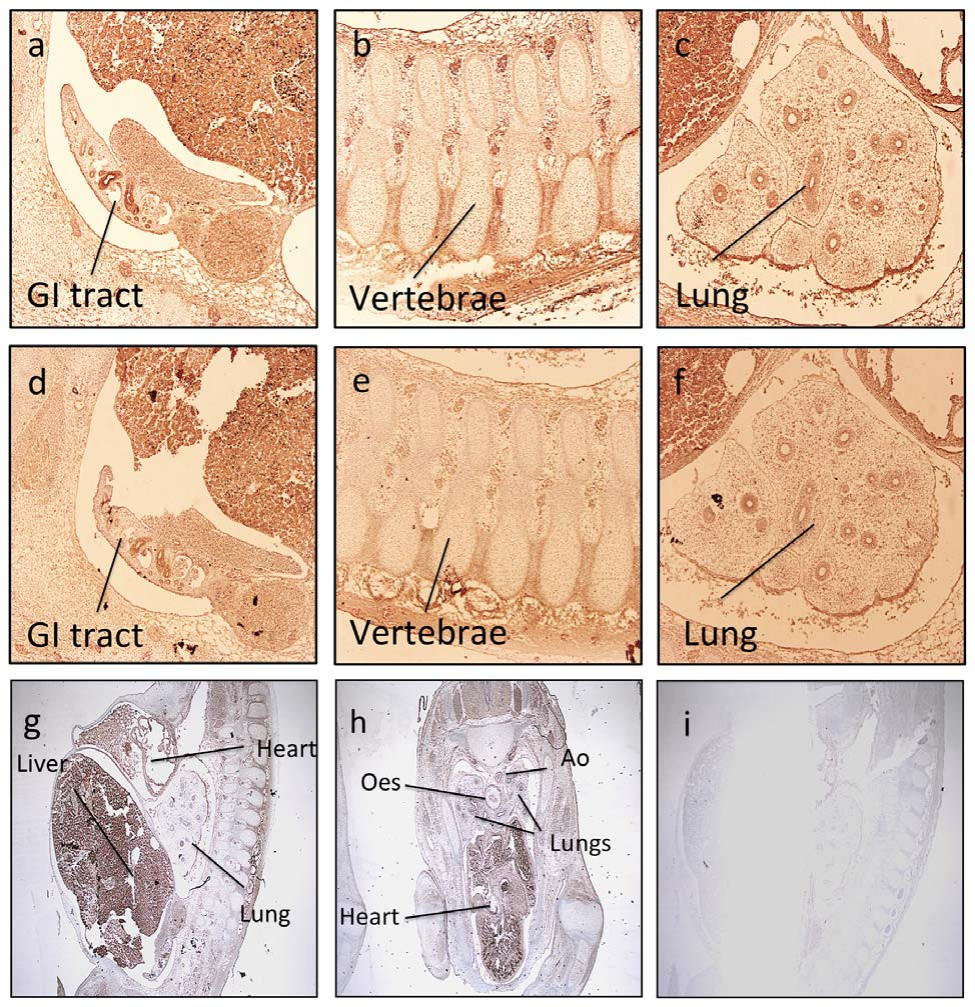
Tissue expression of HOXD8 and HOXD9 in the developing fetus demonstrates cardiovascular expression. (magnificationx100). a–c) HOXD8 d–f) HOXD9. Low power magnification (x40) of g) HOXD8 and h) HOXD9. i) Negative control.

**Figure 10:**
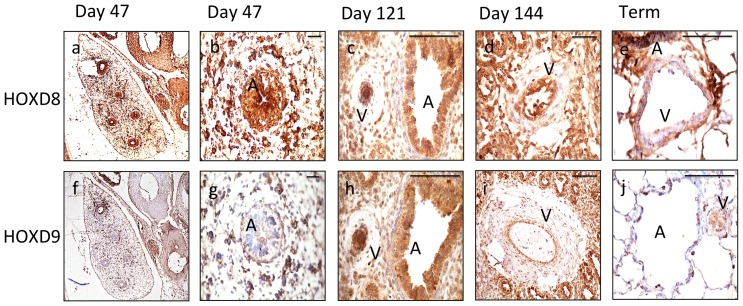
Fetal lung sections from distinct timepoints during fetal development demonstrate endothelial expression of HOXD8 and HOXD9. Expression of HOXD8 (a–e) and HOXD9 (f–j) during embryo and fetal development at 47 days (vasculogenesis), days 121 and 144 (angiogenesis), term and in adult lung. Whole lung day 47 (magnification ×40); developing bronchus/airway with surrounding islands of vasculogenesis day 47 (x100); vessel during angiogenesis (V) with adjacent airway (A), day 121 (x400); at day 144 the vessel has lumenised and is perfused (x200); term (x400) and adult lung (x200). B =  bronchus, V = vessel. Scale bars 100 μm.

Given the mouse ES cell data suggested HOX involvement in angiogenesis programming, we went on to assess expression of HOXD protein in the developing human fetus. In a fetal series, expression of HOXD8 and HOXD9 in the endothelium of the cardiovascular system was widespread (figure 9). The developing cardiac area was rich in HOXD8 and HOXD9 and expression was seen in the major vessels (figure 9g,h). Expression was also observed in haematopoeitic cells, which share a common ancestry with endothelium and in tissues such as neural and GI tract as previously described (figure 9a-f) [22], [23], [24], [25]. Angiogenesis in the lung is prominent during the pseudoglandular and canalicular stages (demonstrated at days 121 and 144). We observed a distinct endothelial expression of HOXD8 and HOXD9 within the developing lung, which coincided, with the known peak of lung angiogenesis (figure 10a–d, f–i). Similar to the adult tissue, epithelial staining was significant, with expression in the walls of the developing bronchi [26]. As with the adult tissue, staining within arteries was specific to the endothelium in all series. By fetal term, the expression of the HOXD8 and HOXD9 was lower (figure 10e,j).

## Discussion

Our studies aimed at understanding the origins of BOECs have provided insights into endothelial positional identity and show that there are context independent differences in the regulatory network of HOX gene expression in these cells. We confirmed and extended characterization of the BOEC, and provide further information about their potential origin, by demonstrating expression of a microvascular-associated HOX transcriptome. That individual HOX genes are involved in vasculogenesis and angiogenesis is well established. However, it remains challenging to study the function of individual HOX genes because of the degree of functional redundancy. Notably very little research has been undertaken in humans. The reliance in the literature on animal models and specifically the early stages of embryogenesis, has perhaps led to an under-appreciation of the extent of involvement of HOX genes in adult endothelial cell identity, and in particular the HOXD cluster described here. HOXA and B loci have previously been recognized as important in vascular biology, with HOXB5 known to regulate the differentiation of angioblasts and mature endothelial cells from their mesoderm-derived precursors, in addition to having a role in angiogenic sprouting, partly through activation of VEFGR2 [27], [28], [29]. HOXB6 and B7, which we describe as differentially expressed in BOECs and mature large vessel endothelial cells, do not have previously defined roles in angiogenesis. HOXB7 is possibly important in the angiogenic properties of myelomas [30] but the near ubiquitous expression of HOXB7 in all endothelial lines (and the relative absence in other cell lines) in our microarray studies suggests a more fundamental role in endothelial biology. An endothelial transcript of HOXA9 is postulated to be a master regulator of EC differentiation from progenitor cells by directly enhancing transcription of VEGFR2 and eNOS [31]. In our analyses the HOX clusters HOXA7-10 and HOXB5-7 were consistently expressed in potentially more developmentally immature endothelial cells (HUVECs, HUACs and in BOECs). This suggests that HOXB5 and HOXA9 are not just important during vasculogenesis/angiogenesis but remain expressed in differentiated endothelial cells.

We describe a novel HOXD dominated transcription signature, which appears to be associated with the positional fate of endothelial cells. This relationship was detectable despite the publically available transcriptomic datasets being originally generated for other purposes, with differing and static culture conditions. We acknowledge that endothelial gene and protein expression can significantly change in culture conditions and therefore the fidelity of these observations across multiple datasets from a number of different groups is surprising, but likely to be robust. From these data we can conclude that the HOX signature of endothelial cells does not relate simply to organ context since the endothelial cells clustered based on the type of blood vessel rather than with cells from the same body segmentation locations. In fact, in the large heterogeneous dataset of cell types, largely of mesodermal origin HOX genes performed poorly in segregating other cell types. This is perhaps not surprising from a developmental viewpoint. Mesodermal cells, once they receive positional signals, manifest plasticity and the ability to be recruited and differentiated locally, rather than being generated in a highly stereotyped branching programme, which is the case for endothelial cells.

**Figure 11:**
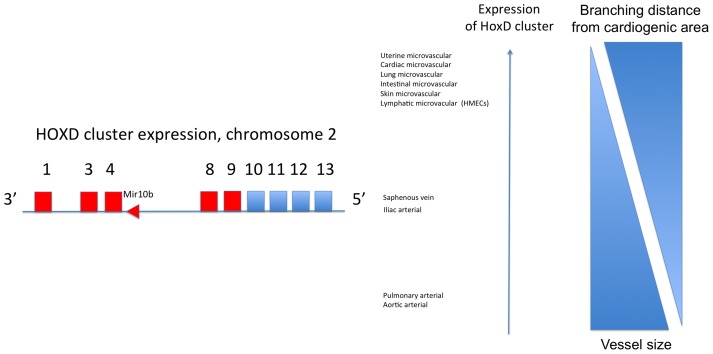
Schemata of HOXD expression patterns according to cellular distance from the cardiogenic area and vessel size.

The demonstration of a similar HOX signature in microvascular cells to those undergoing angiogenesis is of interest as microvascular cells have previously been described as a niche for resident progenitor cells [32]. The position of HOX genes arranged within their clusters 3′ to 5′ along the chromosome is known to be reproduced along the anterior-posterior axis [33]. The mechanism for this remains unclear. In our data the proximal or distal nature of endothelial cells was associated with the HOXD expression pattern (figure 11), and in particular the expression of HOXD8 and D9. Placental endothelial cells have also been reported previously to express a differential pattern of gene expression in genes such as GAX, TLX and DLX between placental distal and proximal cells; genes known to related to, or interact with HOX genes [34]. There is existing evidence that individual HOX genes play important roles in endothelial patterning. For example, neovascularisation through overexpression of Del-1 is mediated by HOXD3 [35]. Angiogenic sprouting is partly dependent on HOXD3 [36] and HOXD3 is expressed in haemoangiomas [37]. HOXD1 has recently been shown to regulate integrin β1 (ITGB1), an important signaling component in angiogenesis [38]. HOXD8 may have a role downstream of Prox1 in small vessel lymphangiogenesis, with vessel diameters significantly larger in a mouse model in which HOXD8 was silenced [39]. Very recently HOXD4 emerged from studies, intended to look at haematopoesis, as a fundamental co-ordinator of vasculogenesis in zebrafish through downstream MEIS-1 [40]. HOXD9 has no as yet elucidated role in endothelial development or function, although the HOXD9 promoter has been described as differentially methylated in sclerosing haemagiomas [41].

The redundancy built into the HOX system, particularly in humans where there are 39 genes in 4 clusters on chromosomes 2,7,12 and 17, makes it challenging to tease out individual contributions of HOX genes to complex processes. Often multiple knockouts of HOX genes in mice are required to see strong phenotypes. We are describing paralogous associations between HOX genes, but perhaps more striking is the clustering of highly expressed HOX genes in chromosomal linear arrangements, and this includes upregulated microRNAs. This is suggestive that in endothelial cells, identity is partly organized along this axis, as in body segmentation specification, and that HOX gene clusters are under the same regulatory control signals. By simply using the primary HOX gene expression pattern, it is remarkable how well the “HOX code” can define endothelial phenotypes in our study. These novel observations provide a basis from which to propose a key role for HOX genes in the positional identity of human endothelial cells.

## Supporting Information

Figure S1 **Adult human tissue staining for HOXD8 demonstrates endothelial staining.** a) Aorta (x200) b) staining of small vessels surrounding the aorta (x630), c) Pulmonary artery (x400), d) small pulmonary arteries (x400), e) small vessels in small bowel (x400), f) bladder (x400), g) stomach (x630), h) placenta (x200), i) skin with microvasculature and epithelium at low power (x200) and j) skin microvasculature at high power (x400), k) small arteries within fat surrounding muscle (x400), l) negative control in pulmonary vessel (x200). Scale bars 100 μm.
(TIF)
