# Supplementary figures and images for: Transcript Analysis Reveals a Specific HOX Signature Associated with Positional Identity of Human Endothelial Cells
Source: PLoS One. 2014 Mar 20;9(3):e91334. doi: 10.1371/journal.pone.0091334 (PMC3961275; doi:10.1371/journal.pone.0091334)

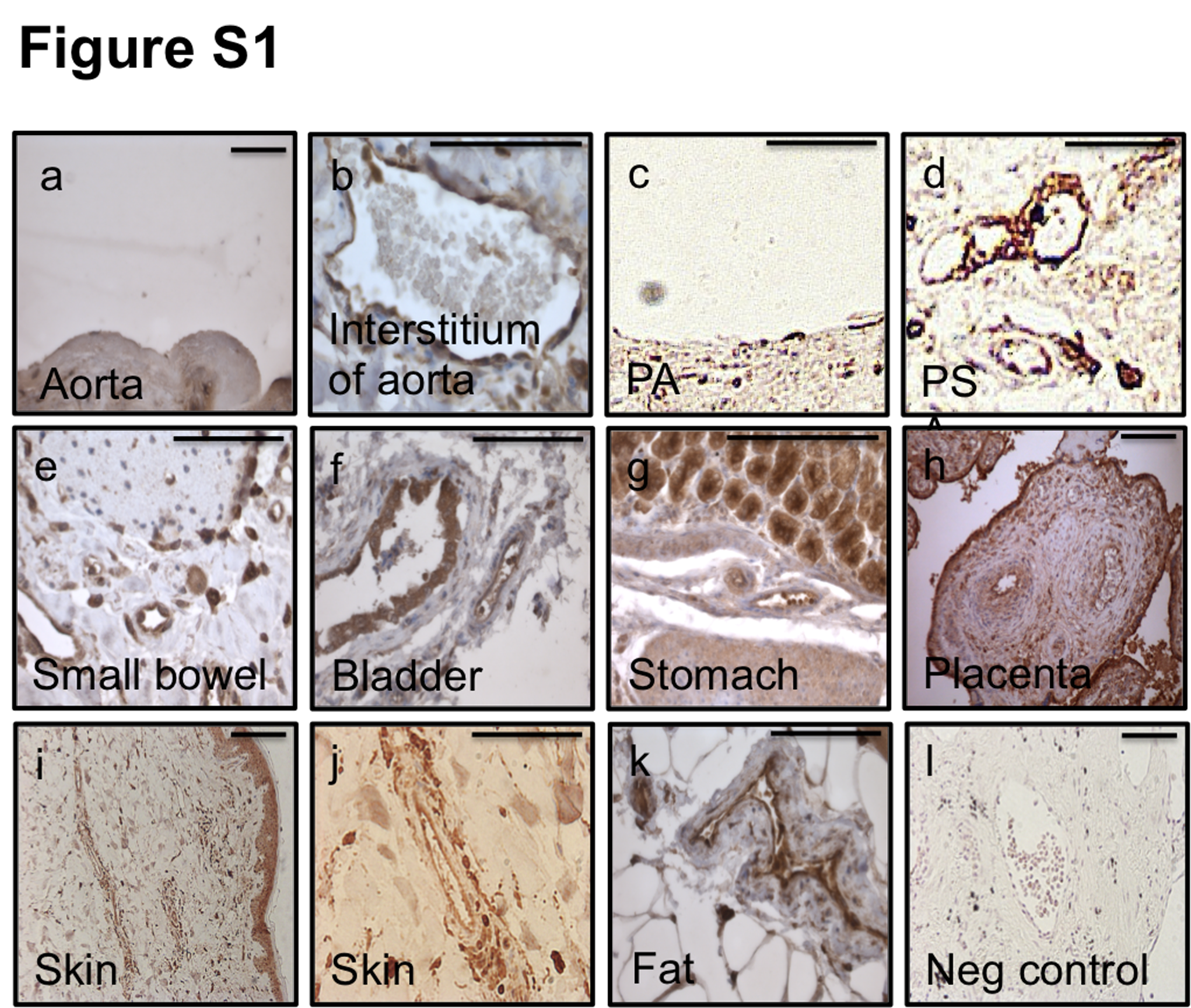

Supplement: Figure S1 — Adult human tissue staining for HOXD8 demonstrates endothelial staining. a) Aorta (x200) b) staining of small vessels surrounding the aorta (x630), c) Pulmonary artery (x400), d) small pulmonary arteries (x400), e) small vessels in small bowel (x400), f) bladder (x400), g) stomach (x630), h) placenta (x200), i) skin with microvasculature and epithelium at low power (x200) and j) skin microvasculature at high power (x400), k) small arteries within fat surrounding muscle (x400), l) negative control in pulmonary vessel (x200). Scale bars 100 μm. (TIF) [file pone.0091334.s001.tif]
